# Layered Seed-Growth of AgGe Football-like Microspheres via Precursor-Free Picosecond Laser Synthesis in Water

**DOI:** 10.1038/srep13661

**Published:** 2015-09-03

**Authors:** Dongshi Zhang, Bilal Gökce, Christian Notthoff, Stephan Barcikowski

**Affiliations:** 1 Technical Chemistry I and Center for Nanointegration Duisburg-Essen (CENIDE), University of Duisburg-Essen, Universitätsstraße 7, 45141, Essen, Germany; 2 Nanoparticle Process Technology and CENIDE, Faculty of Mechanical Engineering & Process Technology, University of Duisburg-Essen, Lotharstr. 1, D-47057 Duisburg, Germany

## Abstract

Hybrid particles are of great significance in terms of their adjustable optical, electronic, magnetic, thermal and mechanical properties. As a novel technique, laser ablation in liquids (LAL) is famous for its precursor-free, “clean” synthesis of hybrid particles with various materials. Till now, almost all the LAL-generated particles originate from the nucleation-growth mechanism. Seed-growth of particles similar to chemical methods seems difficult to be achieved by LAL. Here, we not only present novel patch-joint football-like AgGe microspheres with a diameter in the range of 1 ~ 7 μm achievable by laser ablation in distilled water but also find direct evidences of their layered seed growth mechanism. Many critical factors contribute to the formation of AgGe microspheres: fast laser-generated plasma process provide an excellent condition for generating large amount of Ge and Ag ions/atoms, their initial nucleation and galvanic replacement reaction, while cavitation bubble confinement plays an important role for the increase of AgGe nuclei and subsequent layered growth in water after bubble collapse. Driven by work function difference, Ge acts as nucleation agent for silver during alloy formation. This new seed-growth mechanism for LAL technique opens new opportunities to develop a large variety of novel hybrid materials with controllable properties.

Recently, advance in the synthesis of microscale spherical materials has stimulated their wide applications for super-active catalyst, fast responsive sensor, optoelectronic component, and encapsulation or controlled release of sensitive materials. Different techniques are utilized to give rise to novel microspheres, including sol-gel^1^, steric stabilization^2^, solvothermal reduction^3^. Among them, laser heating in liquid, in terms of its easy setup, one-step process and applicability to various materials, is attracting more and more attention. Till now, it has been successfully applied to generate a large variety of microspheres with high sphericity such as Si^4^, B_4_C^5^, Au^6^, Ag^7^, Fe^8^, ZnO^8^, Cu^8^, CuO^8^, and WO_3_^8^. Under superfluid helium condition with low temperature, ultralow viscosity, high thermal conductivity and chemically inactive environment, it is even applicable to produce single-crystalline semiconductor microspheres with atomic-level smoothness from raw anisotropic materials, such as CeO_2_, ZnSe, CdSe^9^. Some of them have manifested their promise in ultraviolet photodetection^10^ and lubricant oil additive^11^,^12^. What’s more, it also enables to develop spherical hollow TiO_2_ submicrometer microparticles^13^ through a mechanism analogous to the Kirkendall effect^14^,^15^. These size-tailored TiO_2_ hollow spheres with the tunable light scattering ability over a wide visible-light range, once embedded inside quantum dot-sensitized solar cells, can promote a notable 10% current improvement towards the solar-to-electric conversion efficiency. Yang’s group laser-synthesized Ge submicro spheres and manipulated the size from 150 to 600 nm by reducing the liquid layer, thereby showing that both the productivity and the initial Ge concentration in the liquid facilitate the formation of larger Ge particles^16^. In light of the flexibility of the laser-based method to produce functional submicrospheres via heating-melting mechanism^17^, it is interesting to know if the laser technique is able to achieve larger microspheres along the “seed-growth” route like the chemical methods. Thereupon, a review from Grzelczak and Liz-Marzán recently highlights the feasibility of light-assisted synthetic methodologies and announces that “seeded-growth” process may replace the traditional “nucleation and growth” mechanism for the synthesis of metallic nanoparticles^18^. However, currently there is no report on synthesizing nanoparticles using laser ablation on the basis of a “seeded-growth” mechanism. Therefore, considering the unique advantage of versatile laser ablation technique to easily realize ligand- and precursor-free development of novel nanomaterials towards most elements in the Periodic Table^19^ and their embedment in various functional polymers^20^, realization of “seed-growth” mechanism will absolutely provide new inspirations to develop novel “green” and “clean” particles^21^,^22^ by means of laser ablation in liquid for emerging applications, including magnetic resonance imaging (MRI)^23^,^24^,^25^, catalyst^26^,^27^,^28^,^29^, photocatalyst^30^,^31^, photovoltaic solar cells^32^,^33^, surface-enhanced Raman scattering (SERS)^34^, etc.

Herein, we report on the formation of football-like AgGe particles trapped inside laser-induced microholes based on laser ablation of a solid semiconductor wafer in distilled water. Based on the SEM measurements, we propose a layered growth mechanism for the growth of the football-like spherical AgGe particles with different micro sizes. Under high temperature and high pressure condition induced by laser-generated plasma, Ge acts as a good agent for Ag nucleation, together with its solubility in Ag facilitates the formation of AgGe nuclei inside the cavitation bubble and subsequent layered growth of AgGe nuclei after the collapse of cavitation bubbles. The layered seed-growth during plasma expansion or bubble collapse mechanism gives some insights into how Ag and Ge could react in non-equilibrium extreme condition (high temperature, high pressure and supercooling process and grow after bubble collapse), thus may inspiring more hybrid particles to be developed along this seed-growth route.

## Results

**Figure 1:**
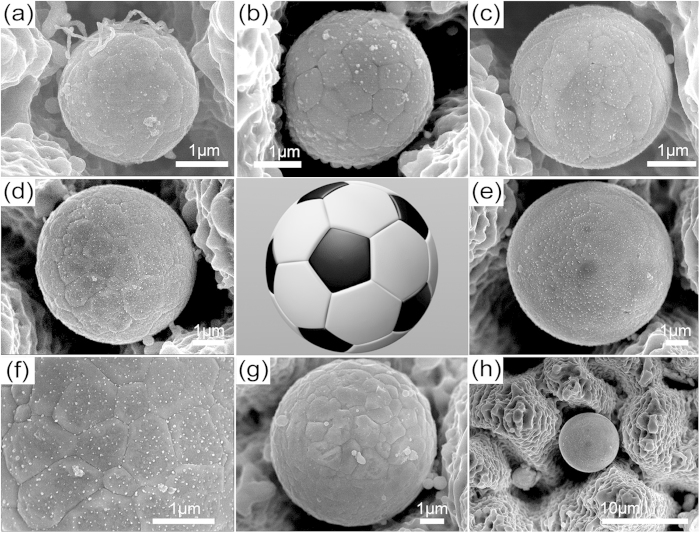
(**a**–**e**,**g**) SEM images of AgGe microspheres captured by laser induced porous microstructures with diameters of 2.5 ± 0.1 μm, 3.3 ± 0.3 μm, 3.6 ± 0.1 μm, 6 ± 0.1 μm, 6.8 ± 0.2 μm, 7.2 ± 0.2 μm. (**f**) Large magnification SEM images of the (**d**) microsphere, (**h**) Micorstructures that capture the microsphere (**e**). *BG designed and created the football figure.*

Figure 1 shows some typical AgGe microspheres captured by laser-induced porous microstructures with different diameters. The microspheres are trapped by adjacent porous microstructures and have diameters in the range of 2.5 ± 0.1 μm to 7.2 ± 0.2 μm. The detailed size distribution of the microspheres captured by the underlying macroscopic surface structures by ten different laser fluences (0.7–12.3 J/cm^2^) are shown in Supplementary Fig. S1. The diameter of the spheres is mainly in the range of 1–3 μm due to the size of underlying microstructures. Meanwhile, some large AgGe spheres are also discovered, with diameters as large as 7.2 ± 0.2 μm at a laser fluence of 5.5 J/cm^2^ (Fig. 1g). Such large spheres are difficult to be developed even through chemical “seed-growth” methods^35^. An interesting phenomenon is the emergence of pentagonal and hexagonal facets on top of the microspheres (Fig. 1f), very similar to the case of ZnO micro-spheres prepared by laser ablation of zinc target in a high-pressure gas atmosphere^36^. Note that the AgGe microspheres and their upper patches resemble the layout of a football, thus we call the spheres “football-like”. Clear observation of the SEM pictures also reveals both the existence of bright clods on top of the microspheres and dots joint to a shell. Such patch-joint football-like AgGe sphere has never been reported before, thereby indicating that a unique formation mechanism may be involved.

**Figure 2:**
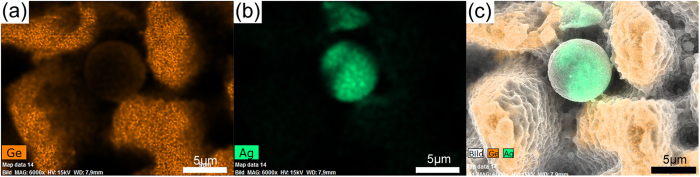
(**a**–**c**) EDX analysis of laser induced synthesized microsphere and porous microstructures achieved at laser fluence of 3.4 J/cm^2^.

Figure 2a–c shows an EDX analysis of the microsphere as well as the microstructures achieved at F = 3.4 J/cm^2^. It is clearly seen that the microbumps are mainly composed of the substrate material, germanium, while in comparison, the AgGe microsphere is mainly composed of Ag with a minority fraction of germanium. After checking some patch pieces from the AgGe spheres (see Supplementary Fig. S2), it is found that different microspheres have different amount of Ag, Ge, C, O, Al elements so that it is difficult to precisely analyse their distribution in all AgGe spheres. Qualitative analysis from four spheres (see Supplementary Table S1) reveals that the average value of Ag in one sphere is 75.88 ± 4.56 wt%, while Ge occupies 21.10 ± 5.32 wt% with O of 1.49 ± 0.31 wt%, C of 1.05 ± 0.40 wt% and Al 0.48 ± 0.33 wt%. To find out which element favors the formation of AgGe microspheres, the debris deposited on the groove achieved at F = 2.3 J/cm^2^ is analysed, as marked by a white circle in Supplementary Fig. S3. The debris is mainly made of Ge, C, O and Al, whereas underneath the debris, no microsphere is found. Therefore, the triggering effect of oxygen, carbon and aluminium on forming AgGe microspheres can be excluded.

**Figure 3:**
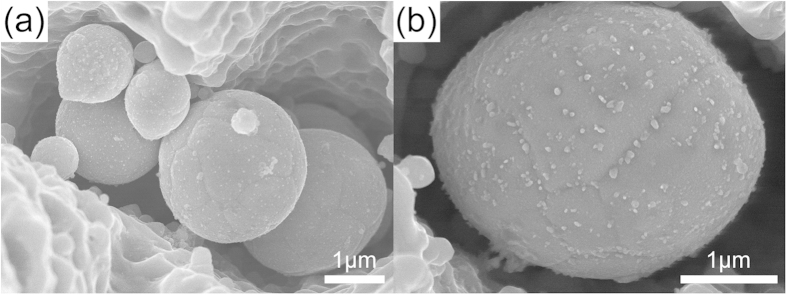
(**a**–**b**) SEM images of stacking and elliptical AgGe particle and captured microstructures achieved at a laser fluence of 3.4 J/cm^2^.

Besides the observation of single AgGe microsphere in one microstructure “trap”, the stacking of many AgGe microspheres and AgGe ellipsoids are also observed, as shown in Fig. 3. The stacked particles (Fig. 3a) with ~1 μm ellipsoid and 2 ~ 3 microspheres indicate that the microspheres do not form in the substrate’s microstructures but form independently after laser ablation before they settle down. This assumption is confirmed by the existence of AgGe particle near the cutting groove (see Supplementary Fig. S4). Figure 3b shows a AgGe ellipsoid with the same layout as the AgGe microspheres as shown in Fig. 1, especially the emergence of the white dots on top of the particles being the same as that of AgGe microsphere shown in Fig. 1f. Compared to the quantity of microspheres, the number of ellipsoids is very limited.

## Discussion

**Figure 4:**
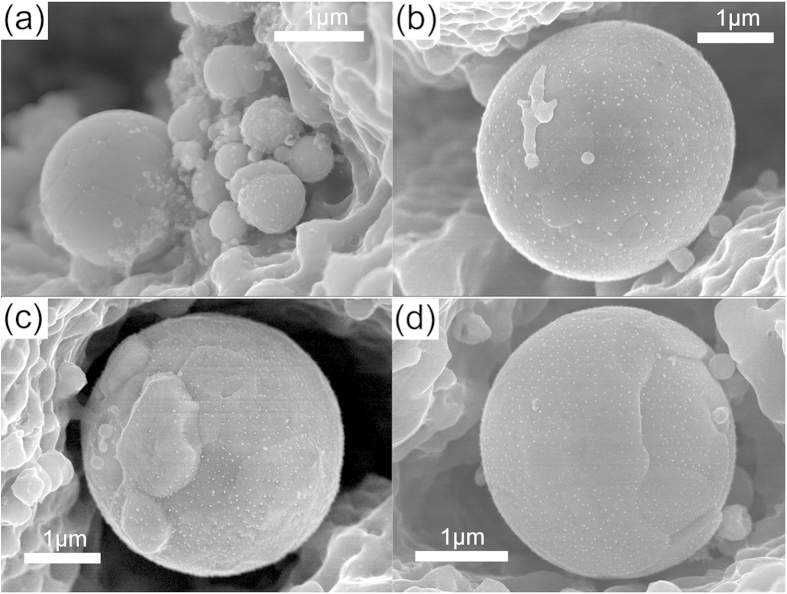
SEM images of the smaller spheres without patches and the growing spheres in different stages to form a layer structure: (**b**) starting growing, (**c**) several “patches” form, (**d**) patches encounter with each other to form one layer. Pictures are taken from different spheres.

Up-to-now, laser induced melting^37^ is still the only mechanism that enables the formation of microscale spherical particles among laser-based techniques. If our results follow this criterion, the particles should be homogeneous inside the particle and smooth in the outmost layer without any chance to show obvious layers inside the particles. But obviously, our results are very different, as indicated by Fig. 1. It is found that some microspheres are composed of different layers with a layer thickness of 100 ~ 220 nm (Fig. 4b–d) which gives direct evidence of the growth of another layer on top of the already formed microscale spheres. Figure 4a shows some smaller spheres (less than 1 μm) and one relatively larger microsphere inside one cavity, as evidenced by the EDX analysis in Supplementary Fig. S5. Only the large sphere has many patches on top of the surface, suggesting that this microsphere is in the submature or mature stage whereas the smaller particles with the size of hundreds of nanometers remain in the embryonic stage. All these findings strongly support that a “seed-growth” mechanism is responsible for the formation of AgGe microspheres, while in comparison, the initial AgGe nuclei should form following the traditional “nucleation and growth” mechanism.

It is well known that seed-growth mechanism generally includes three distinct stages: (1) nucleation; (2) evolution of nuclei into seeds; (3) growth of seeds into nanocrystal of the final microspheres, which is most-likely to be polycrystalline. With regard to the AgGe nucleation, germanium has been proven to be a good nucleating agent for silver^38^. And this nucleation behaviour is non-reciprocal according to the criterion developed by Tiller and Takahashi^39^ based on the difference in their work function (ψ_Ge_ - ψ_Ag_). ψ_Ge _= 4.7 6 eV is larger than ψ_Ag _= 4.30 eV, indicating that Ge phase will nucleate the Ag phase, but Ag phase will not nucleate Ge^38^. This is likely to be the reason why the silver amount is far larger than that of germanium in the AgGe microspheres. Moreover, it has also been found that germanium is soluble in silver and the atomic fraction of germanium in solid solution increases with increasing the temperature^40^. These findings give valuable hints to reasonably explain how these microspheres form during the laser ablation. In addition, nanowire structures of AuGe up to 100 nm in length induced by ns laser ablation of an AuGe alloy in distilled water also demonstrates the possibility to produce interesting alloy structures^41^.

**Figure 5:**
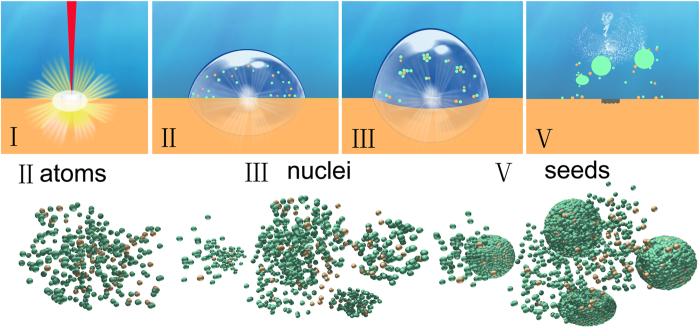
Schematic of the formation mechanism of AgGe microspheres from the perspectives of both macro- (I–V) and the corresponding atom/nano-scale (II–V): I Plasma generation during laser ablation, II bubble generation and dispersed atoms of Ge (orange color) and Ag (green color) atoms. **III** bubble volume increase and atoms coagulate to form nuclei and nuclei grow by coalescence into large particles, **V** bubble collapse and nuclei disperse into liquid as seeds. *DZ designed and created this figure.*

**Figure 6:**
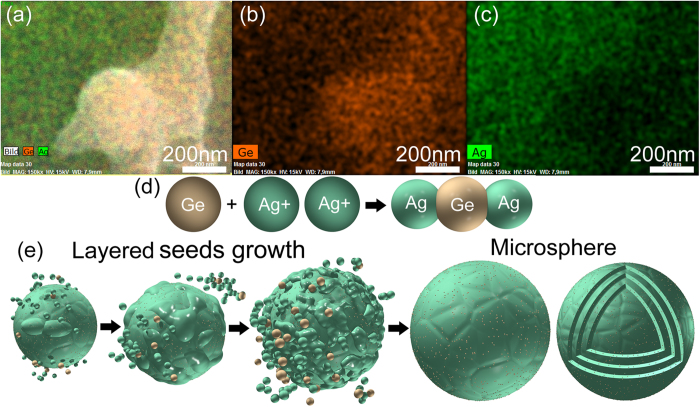
(**a**–**c**) EDX analysis of the growing “clod” shown in Fig. 4b, which has a higher amount of germanium than that of the sphere underneath. This indicates that galvanic replacement between Ge particle and Ag ions (**d**) plays an important role to accelerate the growth of the AgGe microspheres. (**e**) Layered seeds growth mechanism for many seeds to grow into a AgGe microsphere with its cross-section consisting of many AgGe composite layers. *DZ designed and created (***d**–**e**) *of this figure.*

We propose that the seed-growth mechanism for forming AgGe microspheres via laser ablation of Ge target in water should be like the following scenarios shown in Fig. 5. When a laser pulse interacts with the target inside the distilled water, a plasma is generated and accompanied by its induced high temperature and high pressure (Fig. 5I). The temperature (T) could be as high as 6000 K^42^, steadily above the boiling point of Ge (3106 K) and Ag (2435 K), respectively. Therefore, both the ablated silver and germanium materials will be split into atoms/ions (Fig. 5II) inside the laser-generated cavitation bubble. Subsequent adiabatic expansion of the hot plasma will lead to supercooling of the plasma as well as the quenching of both excited elements. De Giacomo and co-workers found that plasma temperature may decrease from ~6000 K to ~3000 K within 60 μs together with the pressure reduction from ~2 × 10^6 ^Pa to ~1 × 10^5^ Pa^43^, indicating that the temperature quenching rate is ~5 × 10^7 ^K/s and pressure quenching rate is 3 × 10^10 ^Pa/s. Such a rapid quenching process provides a favorable environment for ejected particles/atoms to nucleate (Fig. 5III) and evolve into condensate nuclei inside the cavitation bubbles. According to the molecular LaMer nucleation mechanism^35^, once the concentration of the silver atoms reaches a point of supersaturation, they start to condensate and grow to nanometer size. During the coalescence of both germanium and silver atoms into one nucleus, the Ge atoms will serve as “catalyst” for the nucleation of the AgGe alloys and result in the progressive “metallization” of Ge atoms. AgGe nuclei will likely collide with each other to fuse into larger nuclei, thus accelerating the nuclei grow. Due to the local concentration difference in the silver atoms near different nuclei, the growth rate of the AgGe nuclei varies and therefore gives rise to different sizes of AgGe nuclei. Finally, when the bubble pressure is lower than that of the surrounding liquid, the bubble collapses and then leads to the emission of a shockwave with high temperature and pressure. After AgGe seeds disperse into the liquid (Fig. 5V), they will start layered-growth into AgGe microspheres (Fig. 6e). Soon after, many active nuclei adhere to the seeds’ surface by chance. Meanwhile, Ag and Ge dissolve or disperse in these supersaturate seeds’ vicinity and lead to the emergence of more “clods” which expand to cover more surface of the inner sphere till the clods encounter each other and form another intact shell around the growing microsphere. The fast expansion of the AgGe seeds on top of the already formed AgGe spheres may be caused by the galvanic replacement reaction between Ge and Ag^+^ because the growing “clod” in Fig. 4b is Ge-rich (Fig. 6a–c), whereas the microsphere underneath is Ag-rich. This indicates that the growing “clods” must have been oxidized by Ag^+^ during the further growth. The surface of the already formed AgGe spheres serves as an acceptor to absorb Ge atoms/particles and Ag^+^ for the subsequent silver reduction^44^ and Ge oxidization (Fig. 6d). The standard redox potential of Ag is 0.7996 V for the reaction of 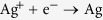
45, while standard redox potential of bulk Ge is −0.124 V for the reaction of 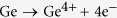
46. The potential of these two half-cells is a positive value (0.6756 V), indicating that a spontaneous occurrence of the following reaction 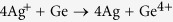 according to the mixed potential theory. This means that galvanic replacement reaction between Ge atoms and Ag ions would accelerate the nucleation process. It is considered that the fusion of the seeds’ “clods” contributes to the appearance of many polygonal facets on the outer surface of the microspheres.

It is well acknowledged that the fraction of Ge atoms dissolved in the AgGe nuclei is limited^47^. Considering 95 percent of Ge in the substrate, the quantity of the Ge atoms/particles generated during laser ablation would be enough for AgGe nuclei to absorb and consume to grow into large microspheres. Due to the limited Ge solubility in Ag, about 15% close to the eutectic temperature^47^, only part of the Ge particles can be taken up by the molten silver. That may be the reason why many bright dots appear on top of each layer rich in Ge. This is in accordance with report that only 4–6 at.% Ge appears in laser-sintering of AuGe nanowires even though the Ge ratio is 26.9 at.% in the target^41^. Meanwhile, it will also cause a strong tendency toward Ag/Ge phase separation as the AgGe spheres are cooled and result in significant precipitates of Ge at the grain boundaries. The grain boundaries are highly mobile and are likely to recrystallize under the used synthesis conditions^48^,^49^, therefore the angles that form at the grain boundary junctions in the polycrystalline AgGe microspheres tend toward 120°^50^ to make the hexagonal and pentagonal features.

Laser ablation in liquid always leads to the formation of pits and ridges^51^, as observed in our case (see Supplementary Fig. S1). Meanwhile, due to the gravitational effect, the already formed microspheres together with many seeds are captured by the “traps” of the underlying periodic microstructures. Note that the AgGe mechanism present here where germanium serves as nucleating agent for metal (e.g. Ag) growth is very different from the metal-catalyzed growth processes where metal nanoparticles (e.g. Ag and Au) react with Ge reagents to yield 1D Ge nanowires^52^ and metal-Ge heterodimers^44^. Considering the strength and diversity of laser synthesis of alloys^53^, this ligand-free layered formation mechanism may pave the way to fabricate more nanomaterials for multiple applications in biology, catalysis and energy fields. These synthesized football-like AgGe particles may possess tunable optical properties for photonic applications^54^.

**Figure 7:**
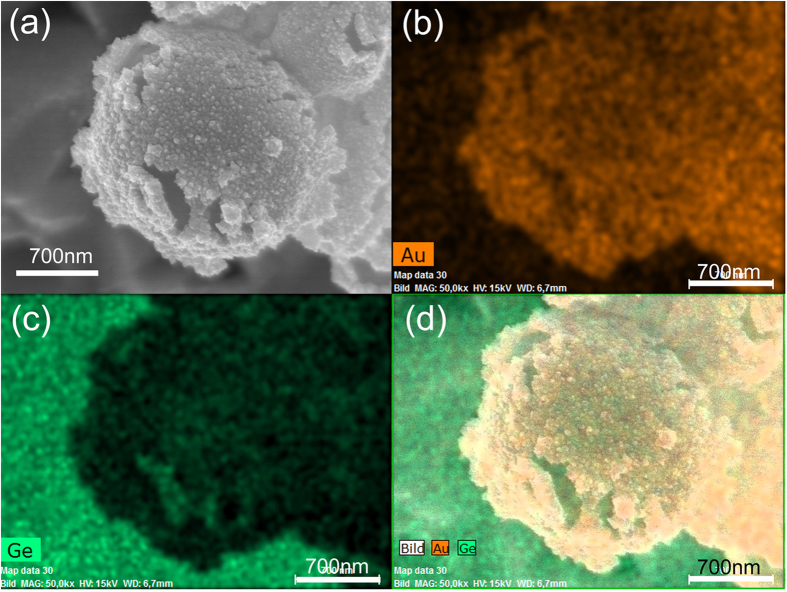
SEM image(a) and EDX images (b–d) of a AuGe microsphere synthesized by galvanic replacement interaction between AuCl_4_^—^ ions and a Ge sphere which is produced by laser irradiation in water.

In order to check whether the preformed Ge seeds could succeed in producing Ag/Au-Ge microparticles, submicrometer spherical Ge spheres synthesized by laser irradiation of germanium powder in water are used to reduce the AuCl4^—^ ions to form AuGe microspheres. The standard redox potential of gold ions is +1.002 V^46^ for the reaction of 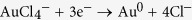. Therefore, Ge spheres are able to reduce AuCl_4_^—^. Fig. 7 displays a synthesized AuGe microparticle with a diameter of 1.2 ± 0.2 μm. The layer thickness is 100 nm ± 10 nm, which is in well accordance with the layer thickness of the football-like AgGe spheres, thereby indicating that the galvanic replacement reaction plays an important role for the formation of the layer structure of the football-like AgGe spheres during laser ablation. It is noteworthy that in using the galvanic replacement reaction: 1) only single layer AuGe microspheres can be generated because the newly formed AuGe layer is mainly consists of Au; 2) no hexagonal and pentagonal features turn out on the microsphere’s surface, which is indicative for the galvanic replacement reaction taking place simultaneously on the whole surface. These findings differ from the layered AgGe microspheres synthesized by laser ablation in liquid where a continuous supplement of melted Ge and AgGe nuclei on top of the preformed AgGe microspheres’ surface is preferable for the seed growth after laser ablation-induced plasma and cavitation bubbles. Meanwhile, it also indicates that the size of the preformed Ge seeds determines the final size of the hybrid particles but not the amount of the seeds. Nevertheless, as for the football-like AgGe microspheres, the amount of Ge particles/atoms and the Ag atoms/ions generated during laser ablation are involved in the seed formation and seed growth and dominate the final size of the synthesized microspheres. In addition, from the perspective of galvanic replacement reaction, germanium is superior to silicon for synthesizing such hybrid microspheres in terms of their different standard redox potential (Ge is −0.124 V, Si is −0.84 V^45^), which means that germanium is much easier to be oxidized to enable the layer growth, while for silicon it is more difficult to reduce gold or silver.

In summary, we prove the feasibility of the laser ablation technique to induce a ligand-free layered seed-growth formation of AgGe microspheres with Ge acting as the assistant nucleating agent. Majority of these microparticles are spherical with size distribution in the range of 1 ~ 7 μm. Minority of them are ellipsoids. SEM images give direct evidence for their unique formation mechanism where germanium is considered to be the crucial factor for the nucleation process as well as the layer growth process under the plasma-induced high temperature and high pressure condition. After comparing one-layer capped AuGe microspheres synthesized by galvanic replacement reaction with laser irradiated Ge microspheres and AuCl_4_^—^ ions, we find that laser ablation allows a continuous supply of large amounts of melted Ge atoms/particles and Ag atoms/ions for their anchoring on top of the formed AgGe seeds. This allows Ag/Ge their further mutual dissolvement and galvanic replacement for the seed growth, thus enabling the generation of more layers and hexagonal and pentagonal features. Overall, this work enriches the diversity of nano/micro hybrid materials achievable by the technique of pulsed laser ablation in liquids. More importantly, it makes an advancement of microsphere formation mechanism, thus paving a way to develop novel “green” microspheres without the need of molecular precursors.

## Methods

Laser ablation is performed in an ablation chamber filled with deionized water (Millipore pH~5–6) using a picosecond pulsed Nd:YAG laser (Ekspla, Atlantic Series, 10 ps, 100 kHz, 150 μJ, 1064 nm). A pump is used to circulate the water during the ablation with the water speed of 6.8 ml/s. The scanning speed of laser ablation is set as 0.1 mm/s and the time interval between two pulses is set as 10 μs. The ablation target is a 200 μm thick germanium wafer (*Dausinger and Giesen*) consisting of 95.28 wt.% of germanium, 1.16 wt.% of oxygen, 1.71 wt.% of carbon, 1.78 wt.% of aluminum and 0.07 wt.% of silver. The XRD result of the grinded powders from the wafer is shown in Fig. S7, indicating the phase’s pureness of the germanium wafer. For laser irradiation, a passage reactor feeds a 1.3 mm capillary at the bottom of a reservoir where the suspension filament is formed into a liquid jet and is irradiated with defined laser fluences^37^. 50 ml colloidal solution with Ge particle mass concentration of 1 g/L in water is used. They are pre-treated in an ultra-sonication bath prior to laser irradiation. A ps laser (Ekspla, atlantic series) is employed with pulse duration of 10 ps, pulse energy of 75 μJ at 532 nm and a repetition rate of 100 kHz. The distance between the center of the liquid filament and the lens (100 mm focal length) is set to be 88 mm. After Ge microsphere synthesis, 0.2 ml colloidal solution is added inside 1.5 ml AuCl_3_ (SIGMA-ALDRICH) and AgNO_3_ (Carl Roth GmbH+ Co. KG) aqueous solution with mass concentration of 1 g/L, respectively. Then the as-prepared hybrid particles after galvanic replacement reaction are dried for SEM analysis. The images showing the morphology and the component variation after laser ablation/irradiation is obtained using a JEOL-JSM7500F cold field emission SEM operated at 15 kV equipped with a Bruker Quantax 200 system equipped with a 30 mm^2^ SSD crystal for the EDX measurement. The penetration depth of the electron beam is 600 nm, which is enough for the beam to pass through the smaller particles with the size of tens of nanometers to obtain the compositions of the spheres. The carbon in the vacuum and the aluminum of the sample holder may induce a small value deviation for the C and Al identification in the AgGe spheres. The size distribution of the microspheres at different laser fluences is analyzed with *Photoshop*.

## Additional Information

**How to cite this article**: Zhang, D. *et al.* Layered Seed-Growth of AgGe Football-like Microspheres via Precursor-Free Picosecond Laser Synthesis in Water. *Sci. Rep.* **5**, 13661; doi: 10.1038/srep13661 (2015).

## Supplementary Material

Supplementary Information
