# Supplementary material for: Layered Seed-Growth of AgGe Football-like Microspheres via Precursor-Free Picosecond Laser Synthesis in Water
Source: Sci Rep. 2015 Sep 3;5:13661. doi: 10.1038/srep13661 (PMC4558578; doi:10.1038/srep13661)
Supplement: Supplementary Information [file srep13661-s1.doc]

**Supporting Information**

**Layered Seed-Growth of AgGe Football-like Microspheres via Precursor-Free Picosecond Laser Synthesis in Water**

**Dongshi Zhang**^1^**,** **Bilal Gökce**^1^**, Christian Notthoff**^2^ **and Stephan Barcikowski**^1^**^*^**

1Technical Chemistry I and Center for Nanointegration Duisburg-Essen (CENIDE), University of Duisburg-Essen, Universitätsstraße 7, 45141, Essen, Germany

2 Nanoparticle Process Technology and CENIDE, Faculty of Mechanical Engineering & Process Technology, University of Duisburg-Essen, Lotharstr. 1, D-47057 Duisburg, Germany

* [stephan.barcikowski@uni-due.de](mailto:stephan.barcikowski@uni-due.de)


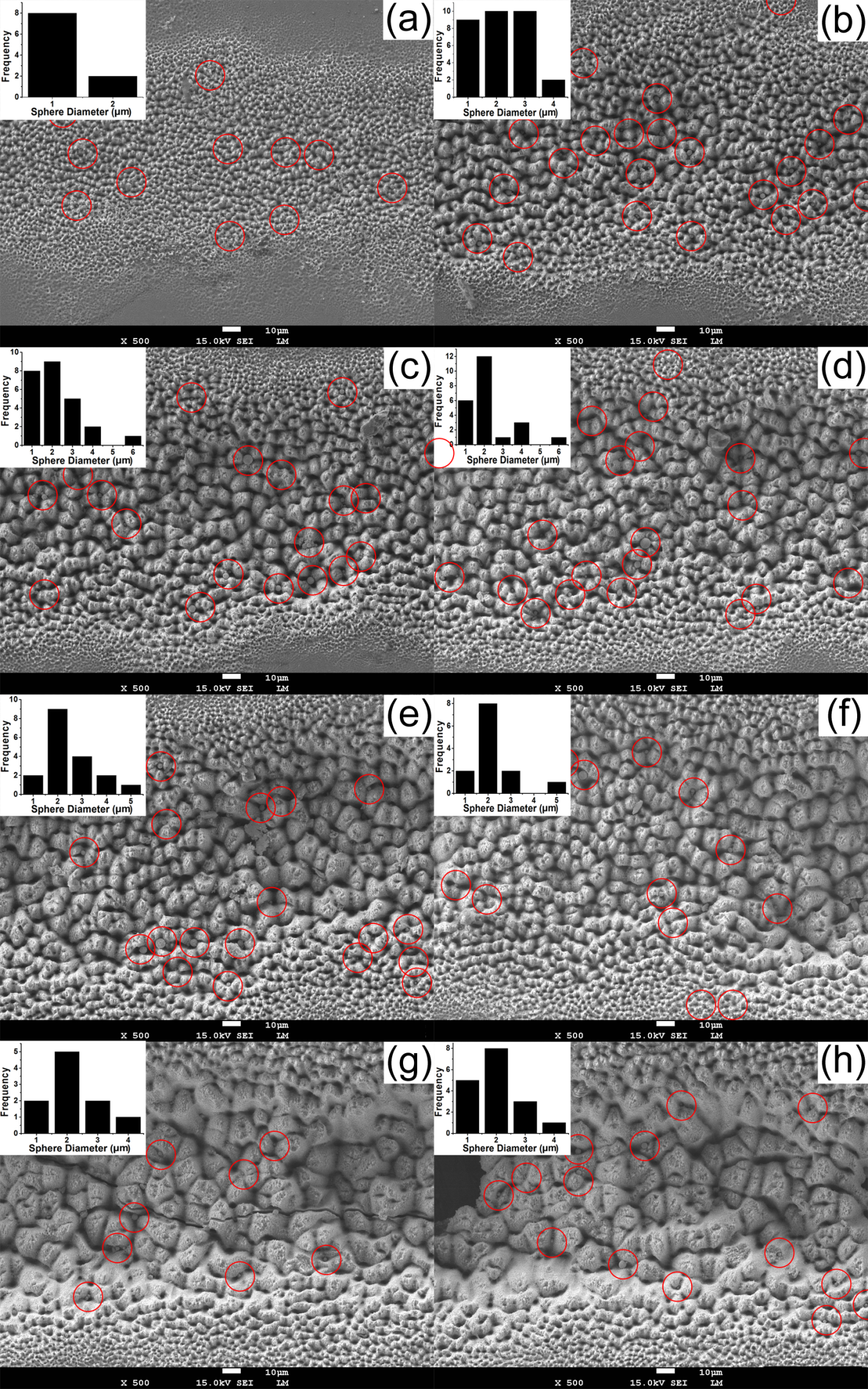


**
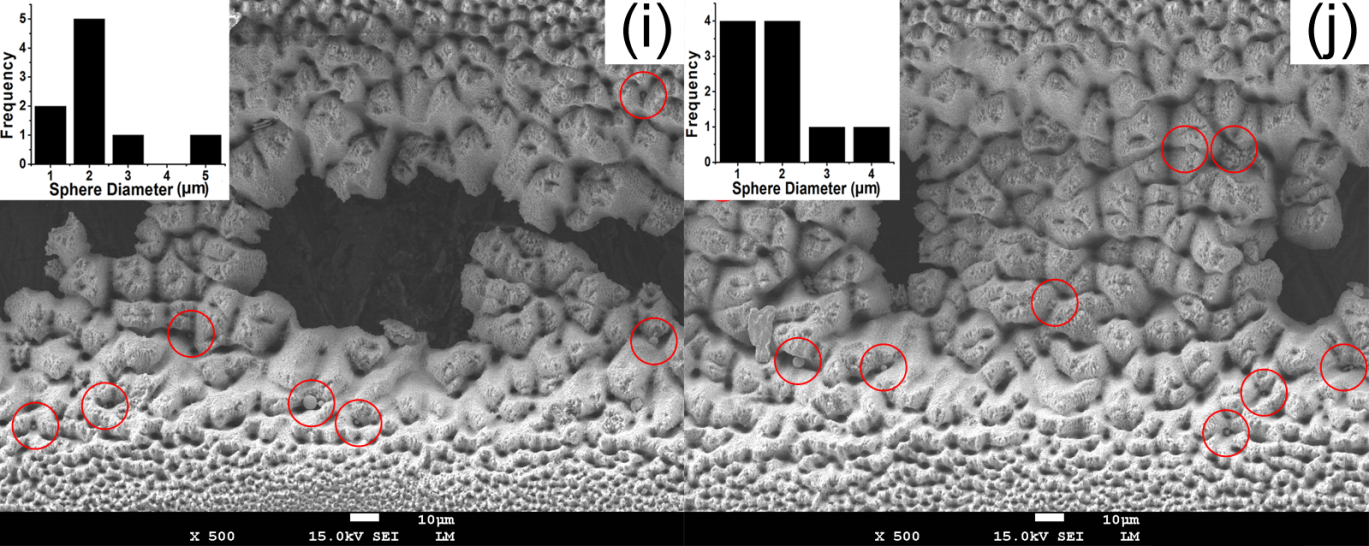
**

**Figure S1** (a-g) The SEM images of the grooves as well as the microsphere distribution at ps laser fluence (F) of 0.73, 2.3, 3.7, 5.5, 7.1, 8.3, 9.4, 10.4, 11.4, 12.3 J/cm^2^ by laser ablation in distilled water. The red circles mark the microspheres captured by the underlying microstructures.

It is found that laser power apparently changes both the morphologies of the cutting grooves and the density of microspheres. At F=0.73J/cm^2^, many microscale protrusions appear on the target surface where subjected to the laser ablation. As the increase of laser power from 0.73 to 12.3 J/cm^2^, small microprotrusions fuse together and evolve into micro bumps with the size in the middle of the grooves changing from ~1μm to ~20 μm. Therefore, the capturing ability of the microprotrusions also varies. At F=0.73J/cm^2^, because of the small gap between the adjacent microprotrusions, the size of the captured microsphere is very small (1-2 μm). With the emergence of large gap between microprotrusions (Figure S1b-d), larger microspheres can be captured, even the diameter up to 6 μm. Further increase in the laser fluence leads to the cut-through of the wafer and broadening of the microprotrusions, both of which will apparently reduce the density of the captured microspheres. The size gradient of the microprotrutions from the center to the edge of the grooves perpendicular to the cutting path for all samples is owing to the energy distribution of the laser beam.

**
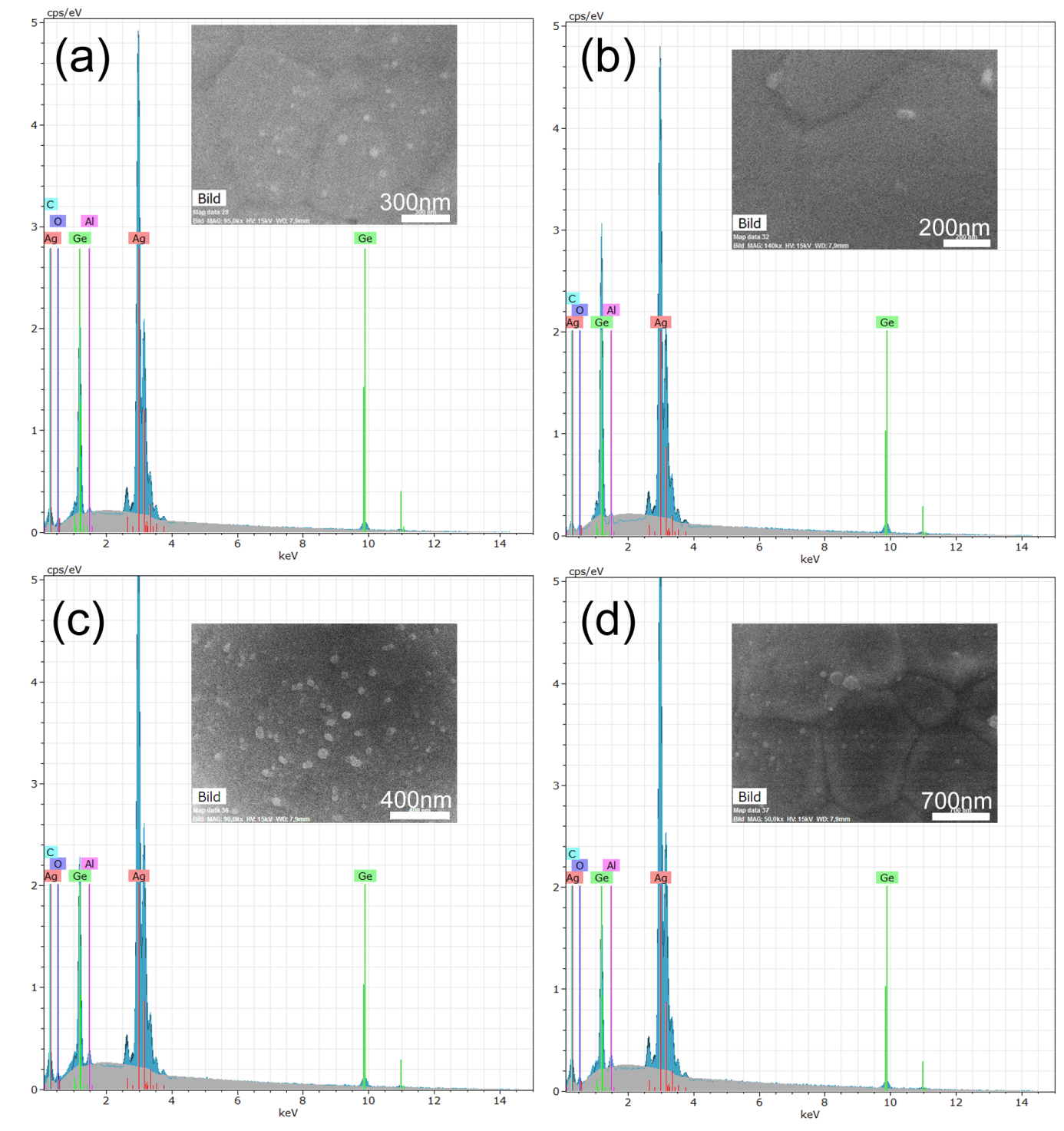
**

**Figure S2** EDX analysis of some patches on four different AgGe spheres with the corresponding SEM image imbedded inside.

**Table S1** Weight percent (wt %) of Ag, Ge, O, C, Al elements measured from Figure S2 (a-d) with respect to four different AgGe spheres and the calculated average value for each element.

|  | Sample a | Sample b | Sample c | Sample d | Average |
| --- | --- | --- | --- | --- | --- |
| Ag | 75.42 | 70.13 | 76.77 | 81.19 | 75.88±4.56 |
| Ge | 21.73 | 28.11 | 19.04 | 15.51 | 21.10±5.32 |
| O | 1.52 | 1.11 | 1.87 | 1.47 | 1.49±0.31 |
| C | 1.03 | 0.55 | 1.51 | 1.13 | 1.05±0.40 |
| Al | 0.3 | 0.10 | 0.81 | 0.7 | 0.48±0.33 |


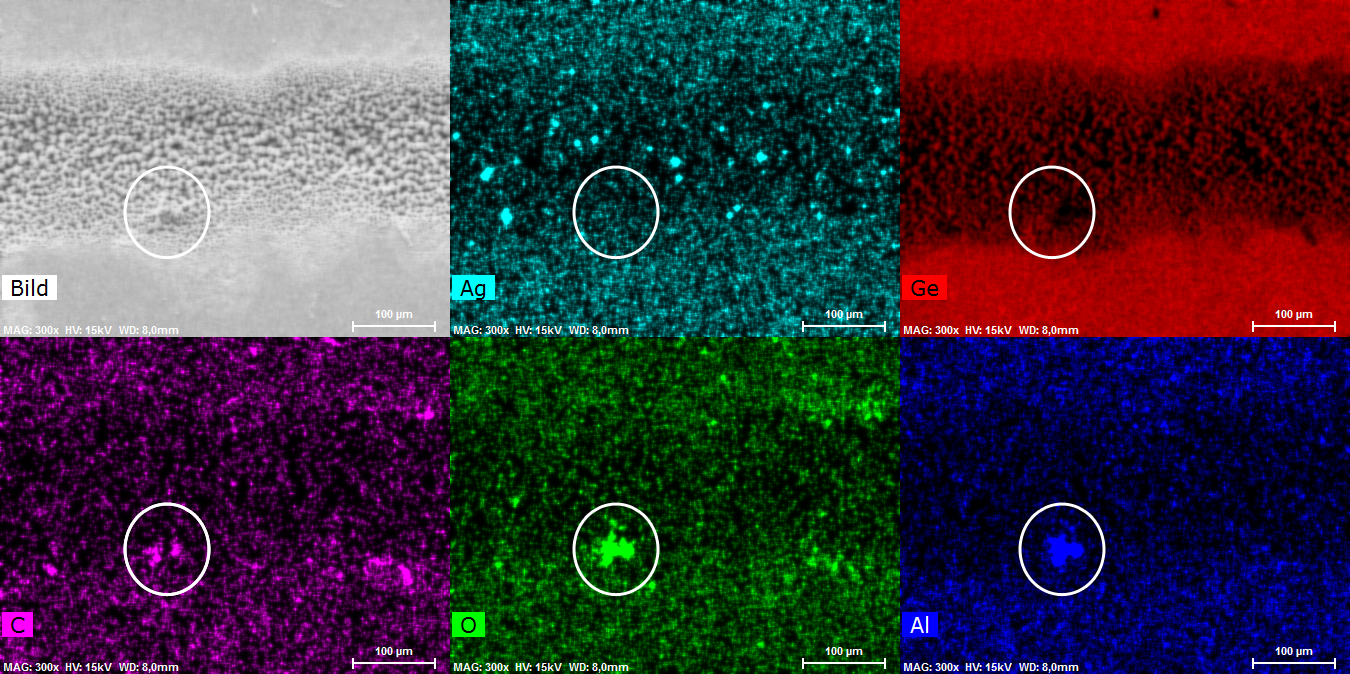


**Figure S3** SEM image and EDX analysis of the stacking microspheres obtained at laser fluence of 2.3 J/cm^2^.


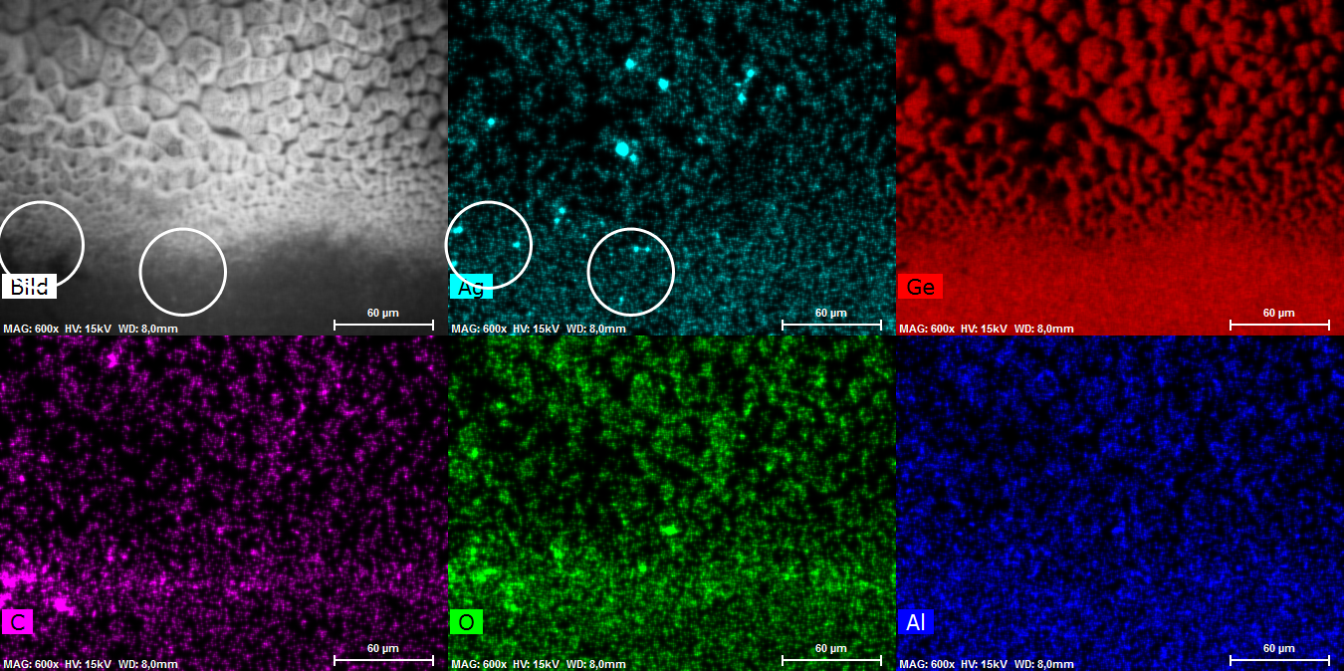


**Figure S4** EDX images of the groove ablated by laser at fluence of 8.3 J/cm^2^.


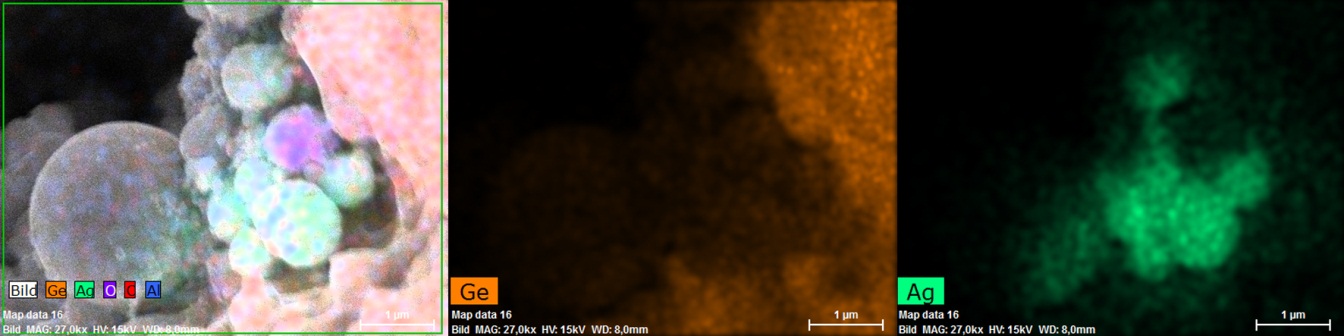


**Figure S5** SEM image and EDX analysis of the stacking microspheres obtained at laser fluence of 7.1J/cm^2^.


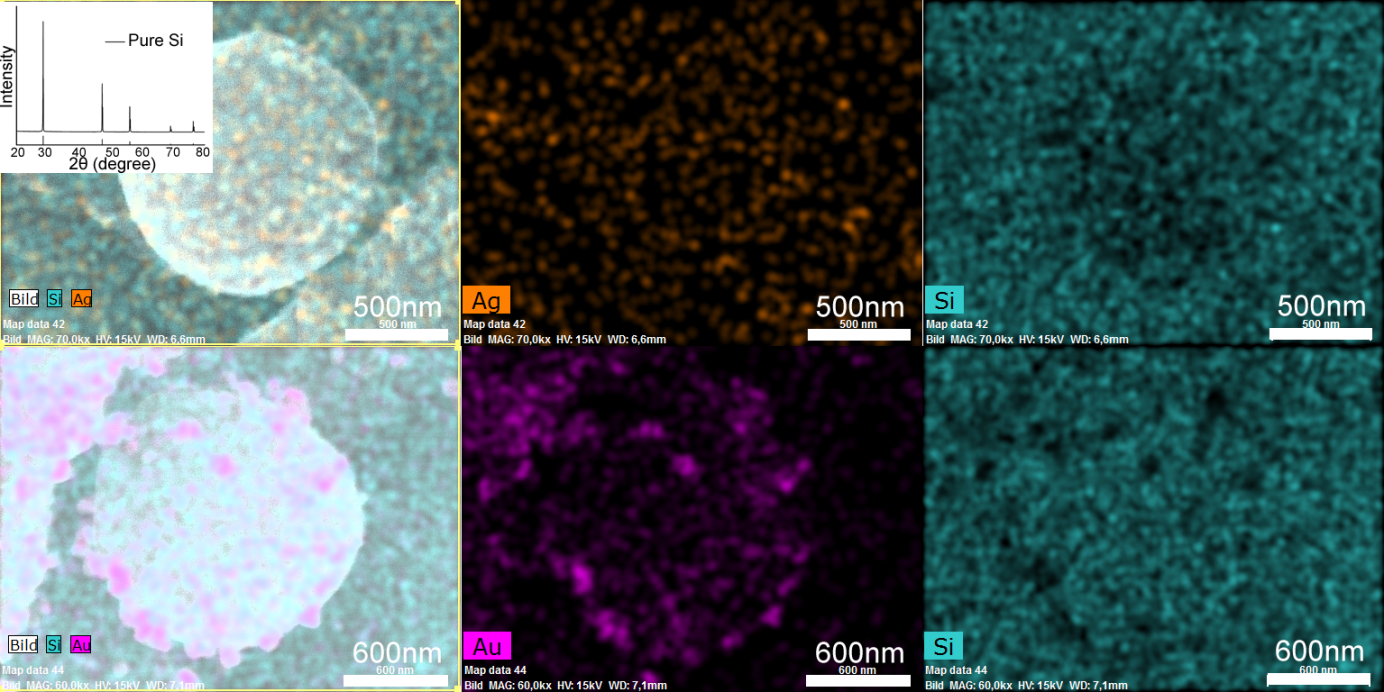


**Figure S6**. EDX images of the silicon sphere decorated by Ag and Au particles after galvanic replacement reaction between Ge and Ag^+^ and AuCl_4_^—^, respectively. The inset figure is the XRD pattern of silicon material, which agrees well with ICSD Coll. Code: 29287.


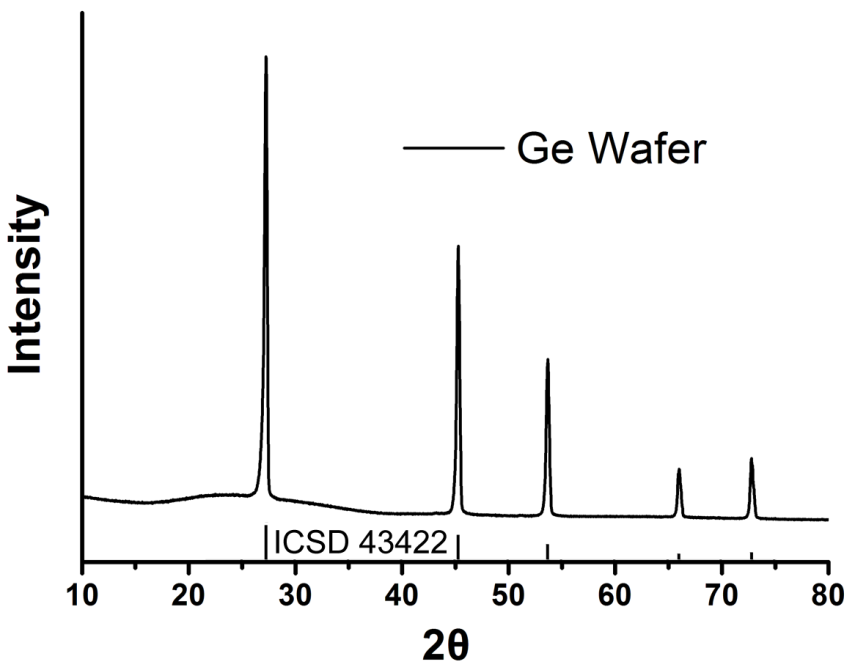


**Figure S7** XRD pattern of Ge substrate, which is in good agreement with ICSD Coll. Code: 43422.
